# Pulse Crop Genetics for a Sustainable Future: Where We Are Now and Where We Should Be Heading

**DOI:** 10.3389/fpls.2020.00531

**Published:** 2020-04-30

**Authors:** Nurul Amylia Sahruzaini, Nur Ardiyana Rejab, Jennifer Ann Harikrishna, Nur Kusaira Khairul Ikram, Ismanizan Ismail, Hazel Marie Kugan, Acga Cheng

**Affiliations:** ^1^Institute of Biological Sciences, Faculty of Science, University of Malaya, Kuala Lumpur, Malaysia; ^2^Centre for Research in Biotechnology for Agriculture (CEBAR), University of Malaya, Kuala Lumpur, Malaysia; ^3^Institute of Systems Biology (INBIOSIS), Universiti Kebangsaan Malaysia, Bangi, Malaysia

**Keywords:** climate change, food security, legume, pulse genetics, sustainable agriculture

## Abstract

The last decade has witnessed dramatic changes in global food consumption patterns mainly because of population growth and economic development. Food substitutions for healthier eating, such as swapping regular servings of meat for protein-rich crops, is an emerging diet trend that may shape the future of food systems and the environment worldwide. To meet the erratic consumer demand in a rapidly changing world where resources become increasingly scarce due largely to anthropogenic activity, the need to develop crops that benefit both human health and the environment has become urgent. Legumes are often considered to be affordable plant-based sources of dietary proteins. Growing legumes provides significant benefits to cropping systems and the environment because of their natural ability to perform symbiotic nitrogen fixation, which enhances both soil fertility and water-use efficiency. In recent years, the focus in legume research has seen a transition from merely improving economically important species such as soybeans to increasingly turning attention to some promising underutilized species whose genetic resources hold the potential to address global challenges such as food security and climate change. Pulse crops have gained in popularity as an affordable source of food or feed; in fact, the United Nations designated 2016 as the International Year of Pulses, proclaiming their critical role in enhancing global food security. Given that many studies have been conducted on numerous underutilized pulse crops across the world, we provide a systematic review of the related literature to identify gaps and opportunities in pulse crop genetics research. We then discuss plausible strategies for developing and using pulse crops to strengthen food and nutrition security in the face of climate and anthropogenic changes.

## Introduction

Publications spanning the past decade paint a grim picture of the future of agriculture, whereby radical measures will be required to sustainably feed the estimated 9–10 billion people by 2050 amid climate change and dwindling land and water resources for agriculture (Godfray et al., 2010; Massawe et al., 2016). The clearing of land for various anthropogenic activities, particularly agriculture, has changed the concentrations of the natural atmospheric greenhouse, causing the Earth to become warmer (Houghton et al., 2012). A stronger greenhouse effect that shifts climate patterns may change the areas where crops grow best, and this would affect the future of crop domestication. Overcoming the adverse effects of anthropogenically driven changes will require tremendous efforts from multiple parties, including researchers from multiple disciplines, policy makers, and the public. Globally, meeting food and nutritional security is already a challenge, as evidenced by the over 800 million undernourished people who face chronic food deprivation (FAO et al., 2018), indicating that food and nutrition security is at stake. Hence, more affordable nutritious foods should be introduced or developed to end all forms of hunger and malnutrition, in line with the aim of the United Nations (UN) Sustainable Development Goals (SDGs) (United Nations, 2015). Within the developing world, dietary protein sources are often limited to cereals due to higher prices of meat (red meat, poultry, fish/seafood, and meat from other edible, managed species) (AMSA [American Meat Science Association], 2017), and the amount of protein consumed is often insufficient in comparison with dietary requirements (Henchion et al., 2017). Furthermore, the refined cereals usually contain mostly carbohydrates and lack important micronutrients such as vitamins and minerals (Sarwar, 2013; Bohra et al., 2015). It is, therefore, important to introduce affordable alternatives to meat and cereals to eliminate the protein gap worldwide. Pulse crops, which represent legume crops that are harvested mainly for their dry grains, may be the most suitable alternatives for a rapidly changing world (Cheng et al., 2019).

Pulse crops are among the best plant-based sources of dietary protein and other nutrients such as iron and of dietary fiber (Kouris-Blazos and Belski, 2016; Maphosa and Jideani, 2017). According to Simpson and Campbell (2015), a plant-based agrarian diet high in fruit or legume fiber generally has greater microbial diversity and can positively influence the levels of short-chain fatty acids, which are important for intestinal health. In addition to their nutritious qualities, legumes provide major benefits to cropping systems and the environment because of their natural ability to perform symbiotic nitrogen fixation (SNF) in their root nodules (Graham and Vance, 2003; Stagnari et al., 2017). The legume family (*Leguminosae* or *Fabaceae*), which has over 750 genera and 20,000 species (Polhill, 1981), is the third largest family of flowering plants after the orchid (*Orchidaceae*) and sunflower (*Asteraceae*) families (Walters, 1960). Pulse crops such as chickpea (*Cicer arietinum*) are the primary source of dietary proteins in many developing countries, for instance in Ethiopia where protein hunger and malnutrition are widespread (Upadhyaya et al., 2011). Similar to cereals, pulses are valuable plant resources used for pasture or fodder (Zewdu, 2004; Atnaf et al., 2015). Given that pulses can be utilized as human food and animal feed while improving soil and environmental health, they are sustainable crops to grow and those with high protein content could potentially be introduced as meat alternative worldwide (Maphosa and Jideani, 2017).

**FIGURE 1:**
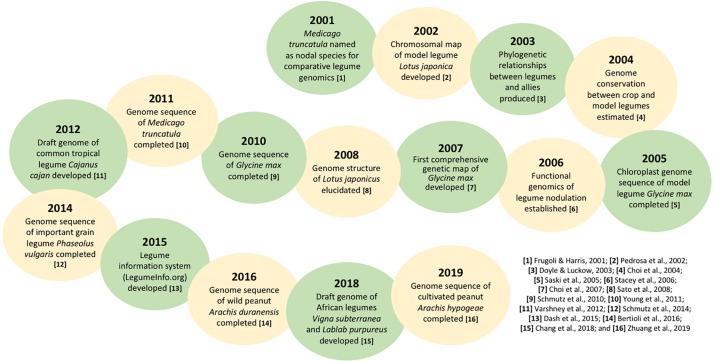
Some major genetic and genomic research milestones for the Leguminosae family since 2000.

The genetic improvement of crops plays a critical role in mitigating climate change and enhancing agricultural systems. Genomic research on commercially important crop species became tremendously active following the completion of The Arabidopsis, and Genome (2000) in the early 2000s, with one notable example being rice (*Oryza sativa*) where a first genome sequence was annotated and published in 2005 (Sasaki and International Rice Genome Sequencing, 2005; Jackson, 2016). In the case of legumes, the genome sequence of the most economically significant species soybean (*Glycine max*) was completed in 2010 (Schmutz et al., 2010). Figure 1 presents some of the major genetic and genomic research milestones for legumes since 2000 (Frugoli and Harris, 2001; Pedrosa et al., 2002; Doyle and Luckow, 2003; Choi et al., 2004; Saski et al., 2005; Stacey et al., 2006; Choi et al., 2007; Sato et al., 2008; Young et al., 2011; Krishnakumar et al., 2014; Dash et al., 2015; Bertioli et al., 2016; Zhuang et al., 2019). During the past two decades, the genetic resources of pulse crops have generally become increasingly available, particularly for a suite of nutrient-dense underutilized species such as lablab (*Lablab purpureus*) and bambara groundnut (*Vigna subterranea*) (Chang et al., 2019). In view of the scattered literature on the advances in genetics of pulse crops, which are the emerging protein alternatives to traditional animal-based food, we provide the first systematic review to furnish insights into key knowledge gaps about pulse genetics which deserve more attention from the scientific community. We then discuss plausible strategies and challenges for the development and utilization of these crops toward strengthening the resilience of the Earth system to climate and anthropogenic changes. We also highlight why we think developing and harnessing the potential of pulse crops can be an ideal solution to food and nutrition insecurity, particularly in the developing world.

## Genetic Resources for Pulse Crop Improvement: a Synthesis of Past Research

Around the mid-1990s, The Food and Agriculture Organization (FAO) of the United Nations created a list of primary pulses and two decades later, facilitated the implementation of the International Year of Pulses (IYP) in 2016. The IYP 2016 was declared to recognize the important contributions of pulses to human well-being and also to the environment. Some examples of these pulses and their available genetic and genomic resources are presented in Table 1. While some of these pulse crops remain underutilized due to factors such as low productivity and limited genomic resources, some of them have recently attracted scientific attention because of their hardiness and nutritional values (Cheng et al., 2019).

**TABLE 1:** Examples of genetic resources in pulses.

Species^a,b^	Genome size (Mbp)^c^	Examples of reported candidate genes and their functions		References
Adzuki bean (*Vigna angularis*)	∼538	*ACT* and *ZMPP*	Waterlogging and biotic stresses	Chi et al. (2016)
		*Fbox*, *UBC* and *PTB*	Salinity-alkalinity and drought stresses	
Chickpea (*Cicer arietinum*)	∼738	*CarLEA4*	Plant development and Abiotic stress	Gu et al. (2012)
		*CarERF116*	Abiotic stress	Deokar et al. (2015)
		Aquaporin family	Biotic and abiotic stresses	Deokar and Tar’an (2016)
Common bean (*Phaseolus vulgaris*)	∼588	*Co*	Anthracnose resistance	Choudhary et al. (2018)
		*CLV2*	Flowering time variation	Raggi et al. (2019)
Cowpea (*Vigna unguiculata*)	∼587	*Dro*	Stay-green	Muchero et al. (2013)
		*Fot3-1*	*Fusarium* wilt	Pottorff et al. (2012)
		MYB113	Black seed coat	Herniter et al. (2018)
Cultivated peanut (*Arachis hypogaea*)	∼2,556	*BjNPR1 and Tfgd*	Enhanced protection against *Cercospora arachidicola* and *Aspergillus flavus*	Sundaresha et al. (2016)
		*AtHDG11*	Salinity and drought stress	Banavath et al. (2018)
Faba bean (*Vicia faba*)	∼13,000	*CHS and DOGT1*	Light adaptation	Yan et al. (2019)
Lentil (*Lens culinaris*)	∼4,000	MIP family	Boron tolerance	Sudheesh et al. (2016)
		NBS-LRR and RLK	Ascochyta blight resistance	Sari et al. (2018)
Mung bean (*Vigna radiata*)	∼540	*VrPGIP1* and *VrPGIP2*	Bruchid resistance	Kaewwongwal et al. (2017)
Pea (*Pisum sativum*)	∼5,000	*DREB2A*	Drought tolerance	Jovanović et al. (2013)
		*MIo*	Powdery mildew resistance	Mohapatra et al. (2016)
Pigeon pea (*Cajanus cajan*)	∼858	*CcTFL1*	Determinacy	Mir et al. (2014)
		*Hsf*	Heat response	Maibam et al. (2015)
		*WRKY* family	Stress tolerance	Singh et al. (2019)
White lupin (*Lupinus albus*)	∼924	*GI, FT* and *SEP*	Flowering time variation	Rychel et al. (2019)

*^a^FAO (1994); ^b^Legume Federation (https://www.legumefederation.org/en/species/); ^c^NCBI, National Centre for Biotechnology Information (available at: https://www.ncbi.nlm.nih.gov, accessed February 19, 2020).*

The symbiotic relationship between legumes and nitrogen-fixing bacteria is known to have a positive impact on both agriculture and the environment. Biological nitrogen fixation (BNF), the only natural means that converts gaseous nitrogen into ammonia and related compounds, plays a pivotal role in soil functions and biodiversity, nutrient cycling, and ecosystem services (El Mujtar et al., 2019; Ferguson et al., 2019). Widening the utilization of legumes while enhancing their nitrogen use efficiency could, therefore, be key to achieving agricultural sustainability (Perchlik and Tegeder, 2017; Ferguson et al., 2019). Several studies have shown that coevolution occurs between some legumes and rhizobia. For example, a study by conducted by Jiao et al. (2018) observed adaptive transcription profiles on different soybean accessions inoculated with rhizobia. The *innB* gene, a novel type III effector of the *Bradyrhizobium* species was found to control the root nodule symbiosis with mung bean (Nguyen et al., 2018; Sugawara et al., 2018).

To date, various molecular approaches have been used to investigate the roles of different legume signals in nodule development. The Nuclear Factor Y (NF-Y) family, for instance, was targeted in common bean and its transcription factors PvNF-YA1 and PvNF-YB7 were reported to be part of a network that enhances nodule formation in the legume (Ripodas et al., 2019). Several genetic and molecular analyses in *L. japonicus* and *G. max* have demonstrated that the Nod factor receptors (NFRs), which mediate Nod factor signals in legumes, are host determinants of symbiosis specificity (Mus et al., 2016). These advances in the understanding of nitrogen fixation within legumes can help to provide avenues for creating non-legume nitrogen-fixing crops through synthetic biology or genetic engineering. Likewise, the completion of *M. truncatula* and *G. max* genome sequencing in the early 2010s has provided significant opportunities to expand the genomic toolbox of other legumes, especially those with more complex genomes (Young and Bharti, 2012; Figure 1).

Environmental fluctuations in the presence of climate change can expose crops to different stress factors, potentially adversely affecting the growth, development, and yield of some species (Varshney et al., 2013; Atieno and Lesueur, 2018). For example, yields of the globally traded legume soybean have been reported to have decreased as much as 40% due in part to biotic and abiotic stresses, including drought, which could also reduce the seed quality of the crop (Githiri et al., 2006; Thao and Tran, 2011). On a positive note, advances in genetic and metabolic engineering over the past two decades have laid a solid foundation for fundamental and applied research in soybeans, especially to enhance its tolerance toward climatic stresses (Yang et al., 2017; Park et al., 2019) and improve its nutritional value as part of devoting significant efforts to securing the global need for biofortified food (Bouis and Saltzman, 2017; Kumar et al., 2019). It is important to note that extensive genetic and genomic analyses have also been undertaken on other leguminous species over the course of the last decade, with an increasing number of web resources related to legume genetics and genomics are becoming available (Table 2). Moreover, recent studies on some promising underutilized pulse crops such as winged bean and lablab have shed light on the genetic basis underlying the growth and development of these species. Thus, the available genetic and genomic resources can be used to facilitate breeding of climate-resilient, improved cultivars through marker-assisted selection (Varshney et al., 2013; Pandey et al., 2016).

**TABLE 2:** List of specific databases and web resources for pulses.

Databases/Web resources	Description	Species	Website
Alfafa Breeder’s Toolbox	A database that provides convenient access to alfalfa genomic, genetic and phenotypic datasets	Alfalfa	https://www.alfalfatoolbox.org
Legume Federation	A database that provides tools/links to various legume genomic resources	Various species including model legumes (e.g., *Lotus japonicus*) and pulses (e.g., peanut)	https://www.legumefederation.org/en/
Legume Information System (LIS)	A collaborative, community resource to facilitate crop improvement by integrating genetic, genomic, and trait data across legume species	Various species including model legumes (e.g., *Lotus japonicus*) and pulses (e.g., peanut)	https://legumeinfo.org
LegumeIP	An integrative database for comparative genomics and transcriptomics of model legumes	Various species including model legumes (e.g., *Lotus japonicus*) and pulses (e.g., peanut)	https://plantgrn.noble.org/LegumeIP/gdp/
Know Pulse	A web-resource focused on diversity data for pulse crop improvement	Various species including chickpea, lentil, dry bean, faba bean, and pea	https://knowpulse.usask.ca
PeanutBase	A web-resource containing genetic and genomic data to enable rapid crop improvement in peanut	Peanut	https://peanutbase.org/
Phytozome	A plant comparative genomics portal	Various species in different plant groups including cereals, pulses, and fruits	https://phytozome.jgi.doe.gov
Pulse Crop Database (PCD)	A database that consists of genomic, genetic, and breeding resources for pulse crop improvement	Various pulse species such as lentil, pea, chickpea, cowpea, bean and others	https://www.pulsedb.org
SoyBase	A database that integrates genetics and genomics to advance soybean research	Soybean	https://www.soybase.org
Vigna Genome Server	Genome server for the genus *Vigna*	Species of the genus *Vigna* such as cowpea	https://viggs.dna.affrc.go.jp

*Source: https://www.legumefederation.org/en/species/.*

## Future Directions and Key Challenges in Pulse Crop Genetic Research

Recent developments in pulse crop genetics and genomics have paved the way for further improvement programs in the years to come. The published genome sequences of multiple pulse crops (Table 1) will enable future exploration to satisfy various research purposes, especially for the preservation of genetic diversity in pulse crops to assure a reserve of variation for future breeding programs. Nonetheless, future advancement of pulse crop research will require more effective strategies to confront a rapidly changing world (Bauchet et al., 2019).

### Plausible Strategies for Pulse Crop Improvement

The substantial reduction in the cost of sequencing that has occurred over the last decade has contributed to the development of several draft genome assemblies for pulse crops such as pigeon pea (*Cajanus cajan*) (Varshney et al., 2012) and common bean (*Phaseolus vulgaris*) (Schmutz et al., 2014). Likewise, it has become a relatively easy and straightforward process to generate transcriptome assemblies, gene predictions, and other sequencing-enabled databases. However, the value of the generated data cannot be fully realized until reliable annotations are performed (Bauchet et al., 2019). As more genetic and genomic resources become available across the Leguminosae family, creating comprehensive resource atlases for all leguminous species is crucial to ease the burden on researchers, especially when large-scale tracing is required. These atlases will be particularly useful for generating gene-based markers that are associated with specific traits and for defining the conserved candidate genes across species as well as those unique to a certain species. The standardization of data storage methods and research frameworks for generating genetic and genomic data should be encouraged.

More focus should be given to constructing pan-genomes to support multiple reference genomes for a single leguminous species such as soybean. Adoption of emerging methods to handle available pan-genomes in a standardized manner is also needed (Bauchet et al., 2019). This perhaps can be achieved by fostering communication among research communities involved in developing pan-genomes for pulse crops. Additionally, a continued effort to understand evolutionary events through interspecific comparative analysis is urgently required. While previous studies have reported that several legume genera such as *Lupinus* (Nelson et al., 2006), *Phaseolus* (Kwak et al., 2012; Guerra-García et al., 2017), and *Vigna* (Isemura et al., 2010; Kongjaimun et al., 2012) have multiple domestications, it generally remains unclear whether the molecular mechanisms underlying these domestication events are shared or species-specific. In the case of underutilized pulse crops, particularly those that represent limited funding opportunities, the capacity to leverage genetic information from well-characterized relatives may prove invaluable.

It is known that accelerated breeding practices are an essential component of crop genetic improvement (Varshney et al., 2014; Watson et al., 2018), which is often measured based on genetic gains per cycle of selection (Bauchet et al., 2019). Although many modern breeding tools and management systems have been actively developed for pulses and food crops overall, some still lack the full functionality that breeders require. For example, even though hundreds of SNP haplotypes have been developed (Lucas et al., 2013; Dwivedi et al., 2017), reliable visualization of these haplotypes for comparisons of closely related entries remains unavailable. Diverse collections of genetic and genomic resources are important for breeders because they archive the diversity on which trait improvement depends (Haussmann et al., 2004; Upadhyaya et al., 2011). Hence, developing better means to manage the widely available current data regarding potential screening materials is essential for breeders in making selection decisions. In other words, easy access to specific data from relevant sources would allow accurate decision-making in a breeding program. In short, further characterization and better maintenance of germplasm collections is imperative to maximize their utility for addressing both current and future threats to pulse crop productivity. With the advances in multiplex molecular assays, it is possible to expand breeding programs that have involved a single targeted trait to multiple traits, for instance, to improve both symbiotic performance and yield of pulse species in different soils.

### Key Challenges for Future Research

Despite the abundance of sequencing data available for pulse crops, data management practices that provide meaningful integration and productive comparison seem not to have kept pace with data generation. By and large, the long-term goal to acquire an improved and shared understanding of legume biology (such as their interactions with rhizobial symbionts, biotic, and abiotic factors) will be difficult to achieve if the ever-growing data are not integrated and remain distributed across multiple databases. The proliferation of biological databases (Table 2) increases the challenge to handle meaningful datasets and raises many questions especially on the quality and reliability of the available resources (Bengtsson-Palme et al., 2016). For example, can we analyze and compare resources from different databases and integrate them into a pan-genome? or what standards of quality should be reconciled when we combine disparate resources so that the analysis will yield insights into underlying biology rather than false positives due to differences in technical approach? Additionally, the quality of the current assembly statistics is insufficiently informative mainly because some of the terms such as “draft” and “reference” are interchangeably used and poorly defined. Ongoing efforts to increase the consistency of assembly and annotation tools and standardize methods for cross referencing metrics would be challenging, as sequencing technologies continue to evolve (Goodwin et al., 2016; Bauchet et al., 2019).

Bioinformatics tools are generally complex with various analysis workflows and applications (Mangul et al., 2019). Researchers often require specific trainings to apply these tools, and one of the biggest challenges is to keep up with the web technologies and frameworks that are constantly changing. Furthermore, most of the tools and databases require ongoing software and security updates, which can be costly and difficult to maintain (Harper et al., 2018). Presently, most of the plant databases store various types of information, from reference genomes to gene or protein annotations and tissue-specific gene expression profiles. Nonetheless, some meaningful datasets remain buried in research papers or only available on membership database which requires a log-in, making it particularly difficult for the research community in low-income and middle-income countries to access the data. Similar to other food crops, future genetic improvement in pulses relies heavily on genetic variation to be used in breeding programs. However, characterizing germplasm and documenting an accurate linkage between an accession and the diverse available sequence information can be challenging, especially when the germplasm collections being genotyped involved some accessions that are highly heterogeneous. Solutions to these multifaceted challenges will require global and regional multilateral efforts.

## A Perspective on Opportunities to Create a More Sustainable Future With Pulse Crops

With an additional 3 billion mouths to feed by 2050, it is questionable whether the current growth rates of agricultural production are sufficient to end global hunger and malnutrition in all its forms. Agriculture is both a leading contributor and a victim of global environmental changes, especially climate change, loss of biodiversity, and freshwater shortages (Ericksen et al., 2009). While animal-based foods tend to be single-source complete proteins, they are often more expensive than the plant-based foods and the high consumption of red and processed meats have recently been reported to be associated with a greater risk of early death (Virtanen et al., 2019). As such, the wide adoption of non-meat protein sources could be one possible means of strengthening a sustainable food future. In recent years, some underutilized pulses such as winged bean and bambara groundnut have been identified as alternative proteins to meat and soybean. These lesser-known species, which are often considered to be “poor man’s crops,” typically contain approximately 20–30% of protein and are rich in certain vitamins and minerals (Singh et al., 2017). With their natural ability to fix nitrogen, legumes can maintain soil fertility and replenish soils that lack nitrogen, making them the most sustainable crops to grow (Stinner, 2015; Stagnari et al., 2017; Kumar et al., 2018).

**FIGURE 2:**
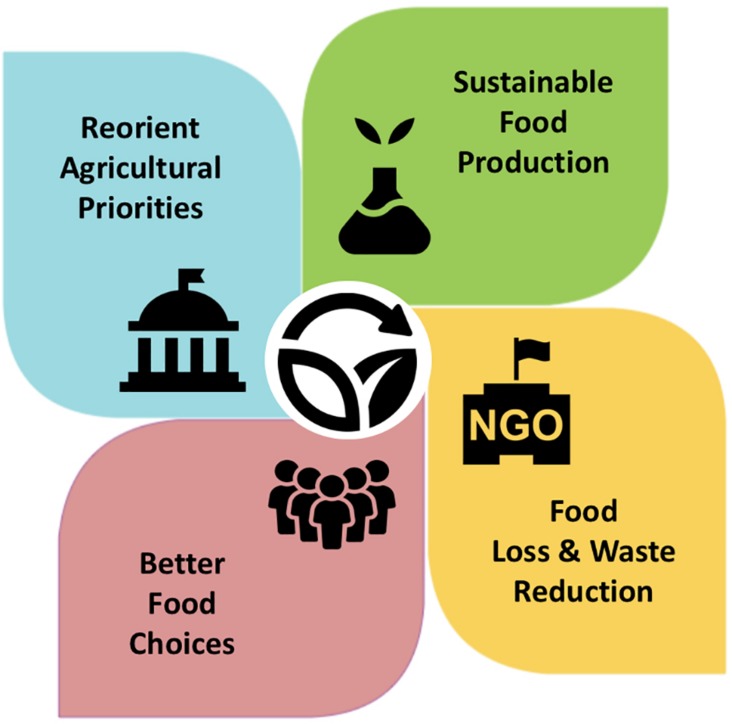
Creating a sustainable food future with pulses: Important strategies and key players.

Increased global grain production over the past half-century has moderately helped in reducing hunger and infant mortality rates in many nations (Patel, 2012; Massawe et al., 2016). While this may denote the success of the green revolution in producing more food, the recent widespread malnutrition and re-emergence of famine in some countries such as Yemen and Nigeria have attracted increasing attention. The solutions to modern-day hunger and malnutrition should focus not only on providing adequate supply of food for all, but also on ensuring equal access to quality and healthful foods (Webb et al., 2018). In fact, food and nutrition insecurity is a multifaceted issue that could be difficult to tackle without collaborative global commitments at all levels of society. Some important strategies and key players to promote sustainable agriculture with pulses are summarized in Figure 2. A range of actions, measures, and policies guided by scientific evidence are urgently required to increase the access to healthy and affordable food, and to bring the United Nations SDGs closer to realization. Ultimately, what humans choose to eat plays a vital role in creating a sustainable food system, because different foods not only have different impacts on human health but also on the environment.

The food and nutrition crisis perhaps can only be resolved effectively by a concerted global effort, involving countries from all levels of development, since the current international food chains are still precariously balanced. Furthermore, many changes in dietary habits and nutrient intakes have been reported in several developed and developing countries (Popkin et al., 2012). For example, traditional diets, which typically consist of more plant-based foods, have been transitioned to the Western pattern diet that generally contains excessive animal-based foods such as meat and dairy. Dietary shift is by and large country-specific, though it is often associated with urbanization and income effect (Holmboe-Ottesen and Wandel, 2012). Recently, the first science-based diet known as the planetary health diet was introduced by the EAT-Lancet Commission to address challenges in maintaining sustainable food systems, which are beneficial to both human health and to the environment. The planetary health diet requires the world to cut the intake of red meat and sugar consumption by half, and to double the intake of pulses, nuts, fruits, and vegetables. This indicates that there is a dire need to develop both traditional and new crops, especially non-cereal crops that have similar strategic advantages in terms of shelf-life such as pulses. Apart from shifting toward a healthful diet, human societies across the globe can perhaps help the world achieve a sustainable food future by reducing food loss and waste.

## Conclusion

With the world’s population slated to reach 10 billion by the mid-century, the transition toward healthier global diets to form sustainable food systems has become an unprecedented challenge. Global warming and anthropogenic activities will make matters worse by initiating land-use changes, which is one of the major drivers affecting soil sustainability and biodiversity. Pulse crops offer varied sources of dietary protein that can be produced more sustainably than animal protein and offer similar storage advantages over fresh produce. It is encouraging that the genetics of an increasing variety of pulses are being explored, as this can assist in sustainable protein production to feed a rising population amidst the challenges created by climate change. However, many challenges lie ahead, and the scientific progress on pulse research should be actuated at both the national and international levels. Addressing knowledge gaps in pulse genetics is essential, and it requires collaborative efforts between researchers and governments to ensure that pulse development and production continue to increase in a sustainable manner. Significant investment into pulse-crop research is essential to revitalize improvement programs in every part of the world, from providing breeders knowledge on appropriate technologies to giving producers improved varieties of grain, particularly for potentially underutilized species.

## Author Contributions

NS, NR, JH, and AC discussed the content. NS and AC drafted the manuscript. NR, JH, II, NK, HK, and AC revised the manuscript. AC acquired the funding. All authors contributed to the final manuscript.

## Conflict of Interest

The authors declare that the research was conducted in the absence of any commercial or financial relationships that could be construed as a potential conflict of interest.
